# Supplementary material for: Case fatality rate and its determinants among admitted stroke patients in public referral hospitals, Northwest, Ethiopia: A prospective cohort study
Source: PLoS One. 2022 Sep 15;17(9):e0273947. doi: 10.1371/journal.pone.0273947 (PMC9477361; doi:10.1371/journal.pone.0273947)
Supplement: Attachment — Submitted filename: Response to reviewers.docx [file pone.0273947.s002.docx]

| S.No | Editor comments | Author Response | Page |
| --- | --- | --- | --- |
| 1 | Please ensure that your manuscript meets PLOS ONE's style requirements, including those for file naming. |  |  |
| 2 | Thank you for stating the following financial disclosure:   [the funders had no role in study design, data collection and analysis, decision to publish, or preparation of the manuscript.]  At this time, please address the following queries |  |  |
| 2a | Please clarify the sources of funding (financial or material support) for your study. List the grants or organizations that supported your study, including funding received from your institution. |  |  |
| 2b | b) State what role the funders took in the study. If the funders had no role in your study, please state: “The funders had no role in study design, data collection and analysis, decision to publish, or preparation of the manuscript.” |  |  |
| 2c | c) If any authors received a salary from any of your funders, please state which authors and which funders. | Thank you, dear editor, no author received salary from the funding |  |
| 2d | d) If you did not receive any funding for this study, please state: “The authors received no specific funding for this work.”  Please include your amended statements within your cover letter; we will change the online submission form on your behalf. |  |  |
| 3 | Thank you for stating the following in the Acknowledgments Section of your manuscript:  [First and foremost we would like to acknowledge participants of the study. We would like to express our deepest gratitude to Debre Tabor University, for funding this research. Our special thanks and appreciation also go to data collectors and staff of the University of Gondar Teaching Hospital, Tibebe Gion comprehensive specialized hospital, and Felege Hiwot referral hospital.]  We note that you have provided funding information that is not currently declared in your Funding Statement. However, funding information should not appear in the Acknowledgments section or other areas of your manuscript. We will only publish funding information present in the Funding Statement section of the online submission form.  Please remove any funding-related text from the manuscript and let us know how you would like to update your Funding Statement. Currently, your Funding Statement reads as follows:   [the funders had no role in study design, data collection and analysis, decision to publish, or preparation of the manuscript.]  Please include your amended statements within your cover letter; we will change the online submission form on your behalf. | Thank you, Dear editor we have made the correction on the acknowledgment section of the manuscript and separately we will send separate cover letter written clearly |  |
| 4 | We note that you have stated that you will provide repository information for your data at acceptance. Should your manuscript be accepted for publication, we will hold it until you provide the relevant accession numbers or DOIs necessary to access your data. If you wish to make changes to your Data Availability statement, please describe these changes in your cover letter and we will update your Data Availability statement to reflect the information you provide. | Thank you, Dear editor we will send the data as supporting fill for you. |  |
| 5 | Your ethics statement should only appear in the Methods section of your manuscript. If your ethics statement is written in any section besides the Methods, please delete it from any other section. | Thank you, Dear Editor we have removed all ethical statements located other than the method section |  |

| S.No | Reviewer comments | Author response | Page |
| --- | --- | --- | --- |
|  | Abstract  Background: a missed word between “was” and “28” | Thank you, dear reviewer, accepted and corrected | Page 2, line 4 |
|  | Results: rewrite this section and organize your findings more efficiently. Perhaps, group the risk factors/determinants into those that increase and those that decrease the 28-day mortality rate. List and properly name the risk factors, so that the readers can understand which sex was the predictor. For example, male sex. Line 14. Please change the wording. The majority (80%) died in hospital and the rest after a discharge from hospital. | We have rewritten the result section |  |
|  | Introduction Line 2. Please, change the wording. By 2020, stroke has become the second leading cause of death. | Thank you, Dear reviewer Comment accepted | Page 3, line 2 |
|  | Please, check the reference N4, African continent is not responsible for 86% of all stroke deaths. It is an overestimation. I believe the correct wording would be that 86% of admitted stroke patients in Africa, die. | Thank you so much accepted and corrected accordingly | Page 3, line 4 |
|  | Inclusion and Exclusion criteria p 12, line 2. Delete admitted patients, keep “All patients diagnosed with stroke and admitted …” | Thank you accepted | Page 5,  Line 2 |
|  | p12, line 3. Change the beginning of the new sentence: We excluded individuals who died before … The exclusion criteria were specified to exclude people admitted with stroke who died before having a CT scan. These were likely the most severe stroke patients. If excluded from analysis, the mortality rates would be underestimated. Please, report how many patients with suspected stroke died on admission before they could undergo a CT scan. Address this in the limitation section. If the proportion is high (15-20%) among all stroke patients who died in hospital, you might want to run a sensitivity analysis adding these individuals. | Thank you for your insight, but we excluded cases not confirmed for two reasons   1. There are cases like brain tumor, hypoglycemia, seizure, brain hematoma, neurosyphilis, complex /atypical migraine and hypertensive encephalopathy ETC which mimic stroke in clinical appearance if we included this case which will lead to over or under estimation of mortality rate 2. If the type of stroke is not confirmed by imaging/CT-scan we cannot compare ischemic and haemorrhagic stroke sub types   So, due to the forementioned reasons we used the confirmed cases  We accept the comment using confirmed cases would under estimate the fatality rate and we included in the limitation section | Page 15 line 9&10 |
|  | p 12, line 13, a comma is missing between education level and alcohol use. | Accepted | Page 5, line 13 |
|  | In the method section: please describe in detail how the 28-mortality rate was calculated, what was used in the numerator and denominator. Why 28 mortality rates? Should it be 28-day case fatality? | Thank you accepted and included.  The reason for selecting is that the mortality rate can be studied at 28 days, 2-month, 6 month and so on but due to resource we used to study the 28 days or acute mortality rate/ more specifically case fatality rate | Page 5, line 26-28 |
|  | Definitions, no definition was provided for how the 28-day mortality rate was calculated, and what constituted the numerator and denominator. Therefore, I cannot clearly understand what rate was reported, however I wondered if a more appropriate terminology to use here would be 28-day case fatality. From what I gathered in the paper, the study included all stroke patients and the outcome of interest was death at 28 days from any cause, the rate was calculated as percentage, so it is a case fatality. | We accept the comment and we made the required changes  We have included operational definition on how stroke fatality rate was calculated | Page 5 line 26-28 |
|  | How did you differentiate between first stroke and readmission for stroke? Did you include only the first hospital admission for stroke into the denominator or any subsequent hospital admission? | Thank you, Dear reviewer, First stroke new onset stroke or if the patient develops stroke after 90 days of previous treatment for stroke |  |
|  | Study variables Please, describe how crude hazard rate and adjusted hazard rate were calculated. How was the adjustment made? | Thank you, Dear reviewer, hazard rate was calculated as death (events) at a time (28 days) divided by stroke patients admitted (at risk of death) as the time approaches to the day 28 from day 1 to day 28 (time approaches to 0)  So, for crude hazard rate the fatality hazard rate was calculated for each variable affecting the outcome variable (no adjustment for covariates) in adjusted hazard rate we included all the variables which were significant on crude hazard rate and we run all the significant variables into the function at a time and the result we get AHR was used. | Page 6 line 1-3 |
|  | Data collection p 13, line 27. I was surprised to read that the data collection was carried out by a GP (general practitioner). Is this correct or was it just a translation issue? Do you have neurologists who review patients with stroke? If this information is indeed correct, you might want to say something more general, for example, “the data collection was carried out by a treating doctor and validated by another physician”, or something similar. This will get you around additional criticism. However, I do have my own reservations about the quality of stroke diagnosis in this study if there was no input from a neurologist or trained stroke physician with data collection. I read in a sentence describing the data quality that a neurologist has assessed the clarity and comprehensiveness of data collected, but how about the quality and accuracy of stroke diagnosis? Have they validated or assessed it in any way? | Thank you, the data collection was carried out jointly by Internal medicine resident (R-3) the confirmation of the diagnosis was made by neuroradiologist, all the other variables were collected by Internal medicine resident (R-3) score like GCS, NIHSS, mRS medication, time to admission, time to initiation of treatment etc. who are trained to treat stroke we used GPs only for the telephone interview for the event (death) occurring after discharge (within 28 days). The role of GPs was only the telephone interview we thought it may mislead the reader and we removed from the manuscript |  |
|  | Table 1. Not the most informative and conventional presentation with these three columns, dead at 28 days and alive at 28 days, and then one for the total population. I would suggest to make a new table 1 with just the descriptive statistics separately for men and women admitted with stroke. This will give a more detailed information about your study population. Then, make table 2 with sociodemographic and other characteristics of men and women who died from stroke at 28 days only. Ideally, you would separate ischaemic and haemorrhagic stroke, as these two types have important differences in risk factors, and particularly, management. I would stress out the importance of presenting the results for specific stroke types, especially given the findings of much higher mortality in patients with ischaemic stroke reported in this study. | Thank you, Dear reviewer we have made separate table, on descriptive statistics of male and females,  But we have included both dead and alive patients at 28 days even if it may not show association, it would be great to show the distribution of factors between the outcomes (dead vs alive), | Page 10 and 11  Table 1&2 |
|  | P 17, line 1. In brackets missing 0.87 |  |  |
|  | P 17. Line 4. The authors reported that mortality was 82% higher among ischaemic stroke patients compared to haemorrhagic. This is an odd finding. There is a general agreement in medicine that haemorrhagic stroke is associated with higher mortality than ischaemic. This is observed in all populations despite their ethnic origins. This is probably the first study to report such dramatic excess in ischaemic stroke death. The possible explanation for the increased mortality rate here is some kind of a misclassification. Perhaps, the type of stroke was not well defined in this study, possibly due to a low quality of the brain imaging, or maybe because of the large proportion of individuals with stroke type not specified, which was actually haemorrhagic stroke. Although, usually cases of unspecified stroke are ischaemic strokes. The authors have mentioned that the stroke diagnosis was confirmed by a CT scan. Do they have access to the MRI or angio? Were all hospitals equipped with the same medical devices? Alternative explanation, might be that patients with severe haemorrhagic stroke don’t get admitted to the hospitals included in the study and were transported to a different facility. I really find it difficult to believe a report that describes such a dramatic difference, unless they can offer a convincing explanation. The current manuscript just states the findings on page 19 without any explanation. However, when I checked the results, in table 2 which reports Hazard Ratios, authors have chosen haemorrhagic stroke as a reference category, and the results showed a lower HR for ischaemic stroke 0.38 (0.2- 0.53). so I am puzzled about why they reported 82%. | We are really sorry, dear reviewer the error occurred when we export the result from STATA to word which leads to mis conclusion. It was unfortunate and we have made the significant correction and rewritten the result and discussion section accordingly.  The stroke types were well classified and confirmed with CT-scan. But the hospitals don’t have MRI or Angio. All the sites we used for data collection were the most equipped in the Northwest Ethiopia. All the three hospitals were equipped with CT-scan, treatment initiation is after confirmation of diagnosis that is why we select these from other hospitals in the region |  |
|  | Page 17, line 6. Instead of “As the Time”, use “If” or “A one hour delay lead to..” The graphs with Kaplan-Mayer survival curves are not numbered and not referred to in the results section. They are just pinned at the end of the text. Please, name the graphs and refer to them in the text. | Accepted, we have made the changes |  |
|  | In the conclusion paragraph, the opening sentence brings up the chronic and infectious disease, but these have not been assessed as comorbidities in the analysis. I would suggest omit the first sentence. Conclusion should be rewritten to provide the summary of the findings, put the findings in the broader context, as well as discuss the implications for public health, research and policy. Next, the authors have discussed the importance of establishing stroke centers and prospective community-based stroke studies, please, omit or explain how these are relevant to the study findings. | Thank you, we have removed the first sentence and rewritten the conclusion | Page 15-line 13-24 |
|  | At the end of the paper, authors have provided a list of acronyms and abbreviations, but did not use some of them in the manuscript. Please, revise the list and delete unnecessary, including HIV, AF, HIV and some other. Second, it would be really interesting to know if stroke outcomes, either survival curves or 28-day mortality rate, were different between HIV positive and HIV negative patients. If the HIV status was known, please, add there results to the text. | Thank you dear reviewer we tried to include HIV status but due to ethical issues we cannot get data regarding the patients HIV status as some of the patients were unconscious |  |
