# Supplementary material for: Case fatality rate and its determinants among admitted stroke patients in public referral hospitals, Northwest, Ethiopia: A prospective cohort study
Source: PLoS One. 2022 Sep 15;17(9):e0273947. doi: 10.1371/journal.pone.0273947 (PMC9477361; doi:10.1371/journal.pone.0273947)
Supplement: Attachment — Submitted filename: 2nd Response to reviewers .docx [file pone.0273947.s003.docx]

| S. No | Reviewer comments | Author response | Page |
| --- | --- | --- | --- |
|  | The authors have made important changes to the manuscript, making it much stronger. However, they still didn’t get the terminology quite right. 1. They have changed 28 mortality rate to stroke fatality rate, which is a wrong term. In their response letter, they have agreed that the reported rate is case fatality rate. However, they are not using the correct terminology in the manuscript. It is important that the authors change the terminology. Since, fatality rate is the death rate observed in a designated series of individuals affected by a simultaneous event (e.g., victims of a disaster). This term should be avoided. – Dictionary of Epidemiology by M. Porta. Instead, use case fatality or case-fatality rate. Some researchers disagree about “rate”, because from a mathematical point of view, by taking values in the range 0 to 100%, it is not a rate, but risk. However, case-fatality rate remains widely used. Check this recent paper: Hatem A. Wafa ,Charles D. A. Wolfe,Ajay Bhalla,Yanzhong Wang Long-term trends in death and dependence after ischaemic strokes: A retrospective cohort study using the South London Stroke Register (SLSR). | Thank you, we have made the necessary change to terminologies! |  |
|  |  |  |  |
|  | Next, some of the findings presented are hard to believe. In particular, that ischaemic stroke had a higher hazard rate of 28-day case fatality than haemorrhagic stroke after adjusting (Table 2). I would strongly suggest that the authors check their data and their model again. In the initial response, they have acknowledged that a mistake occurred with data transferring, which resulted in a wrong conclusion. It is likely that something like this has happened again. Further comments to authors Dear Author, thank you for revising the manuscript, which made it look much stronger. However, I am not completely satisfied with the quality of your paper and would like you to work on it again. These are my comments. |  |  |
|  | Abstract and text – Abbreviations - AHR and CHR. Please, when you introduce these words, first write them in full with the abbreviations in brackets. You can then use the abbreviations. In Table 2 headings, use the words not abbreviations | Thank you, we have used the full terms | Table 3 |
|  | Line 6, page 34. You are making a misleading statement about 86% in-hospital mortality. And using a wrong reference again. Remove reference 2 about stroke and hyperthermia. It doesn’t give information about stroke statistics. Change your statement that Africa accounts for 86% in-hospital death, because it is wrong. I have made this comment before. | Sorry dear reviewer and we have made the necessary changes this time |  |
|  | Line 19, page 34. The sentence needs a reference to support it. The decreased percentage of stroke hospitalization and mortality in developed countries likely reflects the advancements in acute stroke care. You can use the following as a citation Seminog OO, Scarborough P, Wright FL, Rayner M, Goldacre MJ. Determinants of the decline in mortality from acute stroke in England: linked national database study of 795 869 adults. BMJ. 2019 May 22;365:l1778. doi: 10.1136/bmj.l1778. | Thank you dear reviewer and we have included the citation | Page 3 line 16-18 |
|  | Line 4, page 35 Remove Study aim. Replace with Study design and settings | Thank you so much! We have changed the heading | Page 4-line 2 |
|  | Line 11, page 35. Formatting. Remove a full stop | Thank you, removed |  |
|  | Line 12 Remove source and sample size. Replace with Study population. | Thank you, comment accepted | Page 4 line 9 |
|  | Line 15. Remove the number in brackets (554) | Thank you comment accepted | Page 4 line 12 |
|  | Line 16. You don’t need to say that you used the convenient sampling method. | Thank you, comment accepted |  |
|  | Line 10, page 36. Change the dependent variable to 28-days case-fatality rate Type of stroke | Thank you and we have changed to 28 days case fatality rate |  |
|  | . Table 1 type of stroke and Line 6, page 39. Check your data again. There is another error somewhere in your analysis or elsewhere. Around 60% of strokes in your study were classified as haemorrhagic and around 40% as ischaemic. It is very unlikely that you have more haemorrhagic strokes than ischaemic in Ethiopia. Change the sentence “Haemorrhagic stroke was the most prevalent…”, as it is not true. Haemorrhagic stroke is not more common or prevalent in any country. If the results remain the same after you have checked the data and analysis, you must discuss in study limitations why there were more haemorrhagic strokes in your study. Are ischaemic strokes too mild for the admission threshold? Do ischaemic stroke patients go to another hospital for some reason? These are just some possibilities for you to think about. | Sorry dear reviewer, it was typographical error and the problem was not from the data as you can see from table 1 or table 3. And we have checked even the data. | Table 1 |
|  | Table 2. Describe in methods how you did the adjustment of hazard rate? What were your confounders? The adjustment made a big difference to HR by stroke type. The findings that HR for ischaemic stroke was higher than for haemorrhagic are hard to believe. Check your model. | Thank you for your insight. As we checked our data the HR of haemorrhagic stroke was higher compared to ischemic stroke, it is clearly stated in the result and discussion section. Among the methods to control confounder like, randomization, restriction, matching, stratification and multivariate analysis. We used multivariate analysis to handle confounding. If the number of  potential confounders or the level of their grouping is large, multivariate analysis offers the only solution |  |
|  | 14. Line 4, page 42. Change the wording. For example, “We reported 150 deaths from stroke at 28 days among 554 patients hospitalized with stroke. There were 90 deaths were from haemorrhagic and 60 from ischaemic stroke. Next, report CFR for ischaemic and haemorrhagic stroke in men and women separately. Check your data really well. When reporting your findings, do not confuse the proportion of ischaemic and haemorrhagic strokes in admitted patients, and the proportion of these two types in patients who died. The classic scenario would be - more ischaemic strokes on admission (higher %), but more deaths from haemorrhagic stroke at 28 days. So far, your presentation of results is confusing. | We have cleared and rewritten the sentence | Page 8 line 4-9 |
|  | Line 7, page 42. What is the “follow-up stroke fatality rate”? Change this term, as it is not something recognized in epidemiology. Are you talking about death in the community after patient was discharged? | Thank you we have changed it |  |
|  | Line 9 to 13, page 42. Remove this paragraph. It doesn’t provide any useful information, as you are only listing that the variables that you’ve put in the model. | Thank you, dear reviewer we have removed the paragraph. |  |
|  | Table 1. Missing percentages and numbers in numerous places (married female, missing number in the column divorced female, missing % in non-drinker female and missing number in past drinker, etc) | Thank you, dear reviewer the numbers/ figures are there, but when spacing of the tables are rearranged it may disrupt the arrangement of the results. We have put the numbers | Table 1 |
|  | Table 2 and Table 3. Authors have changed the Table 1 to present findings for men and women separately. They should do the same for Tables 2 and 3. This is particularly important, since female sex seems to be protective. | Thank you, dear reviewer, but as our main objective is not to compare based on sex (sex is one factor affecting case fatality rate). We choose to focus on distribution/comparison of factors between patients who were dead and patients who were alive during the follow up time/28 days/ |  |
|  | In the first paragraph of discussion, summarise the main findings. Then, discuss these findings and compare with literature. | Thank you as we accept your comment to have sub heading in the discussion. We will have main finding section and comparison with literatures | Page 10 11 |
|  | Line 4. Remove the first sentence and rephrase the second sentence. You could say this “This is the first study (check if this is correct) reporting case fatality rate for acute ischaemic and haemorrhagic stroke in Northwest Ethiopia. We found that nearly one third of stroke patients dies within 28 days after hospital admission. Case fatality was higher for haemorrhagic stroke (or not) |  |  |
|  | I would advise the authors to use the following subheadings in the discussion. Main findings. Comparison with literature. Strengths and limitations. Conclusions |  |  |
|  | Line 13, page 45. The authors report that 28 day mortality was higher in ischaemic stroke. Not only this sounds wrong, but also contradict their own findings reported earlier, line 4 and line 19, page 42. You reported a 38% higher hazard rate for haemorrhagic stroke. Check your data. Conclusions | Thank you, we have checked our data and the result and the discussions are now in line with our finding. |  |
|  | Line 13, page 46. Instead of listing the variables that you’ve used in the analysis, provide the specific answers. For example, don’t say type of stroke, but haemorrhagic stroke, not sex, but male sex, and so on. This whole sentence might work better in the discussion in main findings section. But I will leave it with you to decide. | Thank you, dear review, it sounds better and we accepted your comments. | Page 12 line 18-23 |
|  | Remove acronyms at the end. | Ok, we have removed |  |
