# Supplementary material for: Case fatality rate and its determinants among admitted stroke patients in public referral hospitals, Northwest, Ethiopia: A prospective cohort study
Source: PLoS One. 2022 Sep 15;17(9):e0273947. doi: 10.1371/journal.pone.0273947 (PMC9477361; doi:10.1371/journal.pone.0273947)
Supplement: Attachment — Submitted filename: 3rd Response to reviewers.docx [file pone.0273947.s004.docx]

| S.No | Reviewer comments | Author Response | Page |
| --- | --- | --- | --- |
| 1 | In the abstract, when you report the findings, please remove the colon symbol “:” when reporting the 95%CI. | Thank you, dear reviewer, accepted |  |
| 2 | Table 3, do not use the asterisk symbols *, **, or *** in the table, as they do not provide any valuable additional information. The 95% confidence intervals provided in the table give all the information that the readers require. Remove the footnotes explaining the asterisks as well. | Thank you, dear reviewer, accepted and corrected | Table 3 |
| 3 | Throughout the manuscript there are words that start with a capital where it should be a lower case. For example, line 9 p 33, neurology ward of the hospitals (Hospital starts with a capital): line 2 page 36, Internal Medicine Resident, it should be all capitals or all lower cases, but not some words. Please, check the manuscript for these typos before resubmitting the final version to the journal | Thank you, comment accepted and corrected |  |
| 4 | In the conclusion section, in the new paragraph that you have added, you have listed all the important determinants that increase and decrease stroke case fatality together. This can confuse the readers. Can you either separate these in two groups, those determinants that were associated with a higher 28 case fatality rate and those that were associated with a lower 28-day case fatality rate? This will make the final part of your paper more clear. Second option might be to only list the parameters that were associated with a lower 28 case fatality rate. Please, correct a minor grammatical error in the last sentence, you can say “Hence, future studies collecting community-based data…" | Thank you we have separated those factors which increase case fatality rate from factors which decrease case fatality rate | conclusion |
